# Assessing the Dynamics of Organic Aerosols over the North Atlantic Ocean

**DOI:** 10.1038/srep45476

**Published:** 2017-03-31

**Authors:** Jérôme Kasparian, Christel Hassler, Bas Ibelings, Nicolas Berti, Sébastien Bigorre, Violeta Djambazova, Elena Gascon-Diez, Grégory Giuliani, Raphaël Houlmann, Denis Kiselev, Pierric de Laborie, Anh-Dao Le, Thibaud Magouroux, Tristan Neri, Daniel Palomino, Stéfanie Pfändler, Nicolas Ray, Gustavo Sousa, Davide Staedler, Federico Tettamanti, Jean-Pierre Wolf, Martin Beniston

**Affiliations:** 1 Université de Genève, Group of Applied Physics, Chemin de Pinchat 22, CH1211 Geneva 4, Switzerland; 2 Université de Genève, Institute for Environmental Sciences, 66 Boulevard Carl Vogt, CH 1211 Geneva 4, Switzerland; 3 Université de Genève, Department F.-A. Forel for Environmental and Aquatic Sciences, 66 Boulevard Carl Vogt, CH 1211 Geneva 4, Switzerland; 4 Woods Hole Oceanic Institute, 86 Water St, Woods Hole, MA 02543, USA; 5 TIBIO SagL, via alla Valle I I, 6949, Switzerland

## Abstract

The influence of aerosols on climate is highly dependent on the particle size distribution, concentration, and composition. In particular, the latter influences their ability to act as cloud condensation nuclei, whereby they impact cloud coverage and precipitation. Here, we simultaneously measured the concentration of aerosols from sea spray over the North Atlantic on board the exhaust-free solar-powered vessel “PlanetSolar”, and the sea surface physico-chemical parameters. We identified organic-bearing particles based on individual particle fluorescence spectra. Organic-bearing aerosols display specific spatio-temporal distributions as compared to total aerosols. We propose an empirical parameterization of the organic-bearing particle concentration, with a dependence on water salinity and sea-surface temperature only. We also show that a very rich mixture of organic aerosols is emitted from the sea surface. Such data will certainly contribute to providing further insight into the influence of aerosols on cloud formation, and be used as input for the improved modeling of aerosols and their role in global climate processes.

The direct and indirect radiative forcing of the atmosphere by aerosols is recognized as a major source of uncertainty in global climate modeling^1^, as highlighted by the recent IPCC reports^2^. This effect is highly dependent on the particle size and composition^3^. Besides the terrestrial aerosols of anthropogenic origin (linked to fossil-fuel combustion, industrial processes, transport…) or those of natural origin (e.g., sand, pollen, bacteria, as well as particles emitted by erosion, fires, volcanoes…), it is now recognized that sea spray aerosols (SSA) are also key players in the climate system. In particular, they are now considered to be the main contributor to light scattering in the marine boundary layer and possibly the most important natural aerosol component capable of influencing the Earth’s radiative balance^4^,^5^.

The production of SSA is closely linked to air bubbles generated in the ocean by breaking waves, which then rise to the surface and burst into hundreds of droplets over a broad size range, from the nm scale to tens of micrometers^6^,^7^. The process efficiency (i.e., the emission flux varying from 10^4^–10^6^ particles/m^2^/s) is primarily linked to wind speed, which modulates the amplitude of oceanic waves and to sea surface temperatures (SST)^8^.

Oceans are one of the largest reservoirs of organic carbon on Earth, with a wide variety of substances^9^. Besides sea-salt, the composition of the aerosol particles is complex^4^,^10^, as it comprises sulfur compounds that can be found in the form of dimethyl sulfide (DMS) in secondary particles^11^, polysaccharides, proteins, amino acids, fatty acids, humic substances, gels, micro-organisms (e.g., phytoplankton or bacteria) and their fragments, as well as vesicles^12^. These active species, together with sulfates^13^,^14^,^15^ can act as cloud condensation nuclei (CCN) and ice crystal nucleating agents in clouds^16^. They therefore contribute indirectly to the planetary radiative balance, in addition to their direct effect via light scattering.

Numerous laboratory studies and marine measurement campaigns have been carried out to identify the relationship between ocean surface characteristics, biological activity and the composition of SSA. No definitive conclusions have so far been reached, due to the complexity of the physico-chemical processes involved, including the aging of aerosol particles. For example, the use of Chlorophyll *a* (Chl *a*) maps retrieved by satellite imagery over the ocean, as a proxy for the concentration of organic aerosols concentrations, is still the subject of intense scientific debate^17^,^18^.

In this paper, we report the results of a large-scale measurement campaign performed over 5,000 nautical miles in the North Atlantic Ocean (from Miami on June 8^th^ to London on August 30^th^, 2013), focusing on the northern part of the campaign, namely the Boston – Halifax – St John’s route (July 4th to August 1st, 2013, See Table S1). This segment of the journey covered a wide range of weather and ocean conditions, with incursions into both cold- and warm-water masses on the margins of the Gulf Stream. The use of the world-largest solar-powered vessel, the Swiss “Tûranor PlanetSolar”^19^, which does not produce exhaust fumes, significantly limited contamination of the measurements. Together with the aerosol particle size, a single-particle fluorescence spectrometer (GAP-SPFS, named after ref. 20), specifically developed for the expedition, continuously characterized the composition of individual particles in real time and *in-situ*. In particular, it allowed to specifically identify organic-bearing particles.

We show that the abundances of total and organic-bearing aerosols, respectively, display different spatio-temporal distributions, and can be modelled by different empirical relationships as a function of the physico-chemical parameters of the ocean and the atmosphere (temperature, wind, salinity). Surprisingly, the organic-bearing particle concentration can be parameterized with a dependence on water salinity and sea-surface temperature only. Furthermore, the fuorescent spectra of individual particles reveal that a very complex mixture of organic aerosols is emitted simultaneously. Such data will provide new insight into the dynamics of organic aerosols and their influence of aerosols on cloud formation.

## Results and Discussion

### Sea spray particle concentration and size distribution

**Figure 1:**
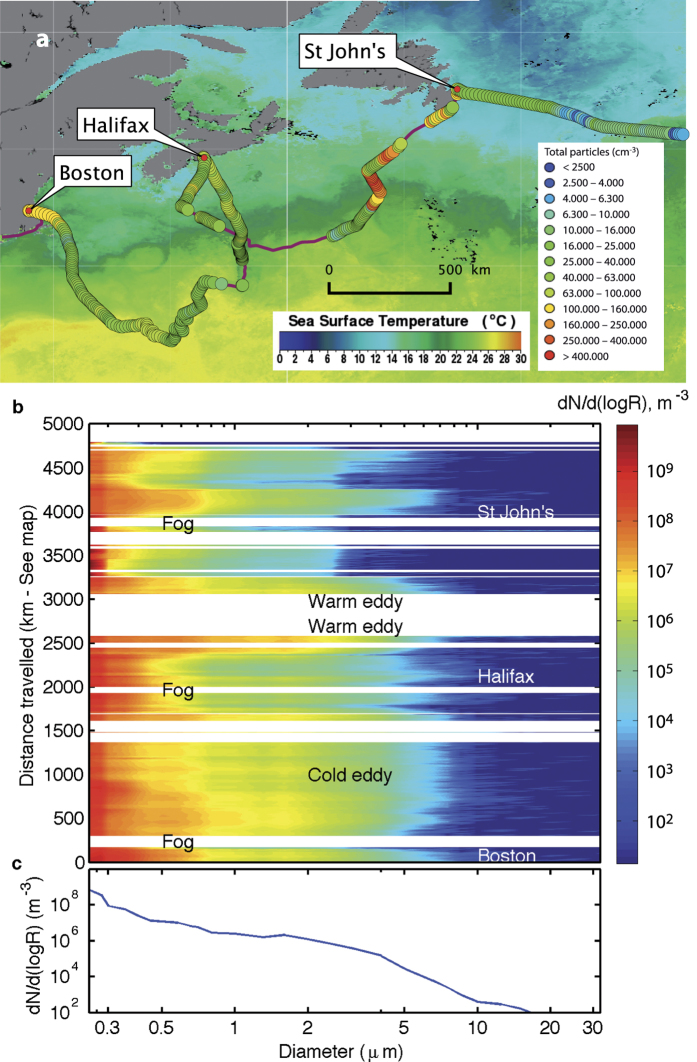
Concentration and size distribution of Sea Spray Aerosols. (**a**) Total particle concentration (>1 μm diameter) and SST (background map, data from AQUA MODIS^26^) between Boston and the open ocean east of St John’s, Newfoundland. Map created with QGIS 2.12^27^ (**b**). Evolution of the size distribution of the detected aerosols as a function of the distance travelled by the ship. (**c**) Cruise-averaged aerosol particle size distribution.

The total aerosol concentrations in the 250 nm−32 μm diameter range varied between 2 × 10^6^ and 5 × 10^8^ m^−3^, with an average of 1.1 × 10^8^ m^−3^ and a median of 1 × 10^8^ m^−3^ (Fig. 1a). Considering a particle lifetime of 5 days^4^ and a mixing layer thickness of 1 km^4^, and integrating over the measured size distribution (Fig. 1c), these concentrations yield a global emission flux of 1,000 Tg aerosols/year. Assuming 5% carbon content as proposed by^4^, this flux corresponds to 50 Tg C/year. Such values are consistent with typical global estimations relying on satellite-based measurements and global model simulations, that yield values ranging from 2 to 300 Tg C/year^4^,^21^,^22^,^23^.

The concentration of particles up to 10 μm was in particular positively correlated with air and sea surface temperature (Fig. 1b; See also Table S3 and Figure S3), while the shape of the distribution seems less affected (Fig. 1c). This evolution might be related to the influence of temperature on the process of bubble fragmentation as suggested from laboratory experiments^24^,^25^. However, methods for simulating actual wave breaking usually require large infrastructures with wave paddle generators or large wind tunnels, which was not the case of the said laboratory experiments. Further experiments are therefore needed to confirm this assumption.

Also, Fig. 1a displays sharp transitions in the particle number concentration, illustrating the local dynamics induced by the contrast between water masses and weather conditions. Furthermore, coastal regions (close to the stopovers in Boston, Halifax and St John’s) are associated with both higher total particle concentration and smaller particles, with a narrower size distribution.

Obviously, particle transport and possible import of terrestrial aerosols have to be considered. However, several observations allow us to expect a moderate influence of land and aerosol transport in our measurement, except close to the stopovers: (i) the fast decay of the number concentration of particles as a function of the distance from the coast; (ii), the lack of correlation between the wind direction and our measurements, and (iii) the dominant wind direction (mostly from the South, i.e., from the open ocean), that was aiming at land all over the considered period, except for 36 hours. We can therefore expect that the data evaluated as “micrometer sized sea spray related” in this paper are not significantly altered by terrestrial imports. Note that, moreover, freely flowing aerosol particles are directly aspired from the atmosphere, so that the true size of the particles/droplets is measured rather than their dry size.

**Figure 2:**
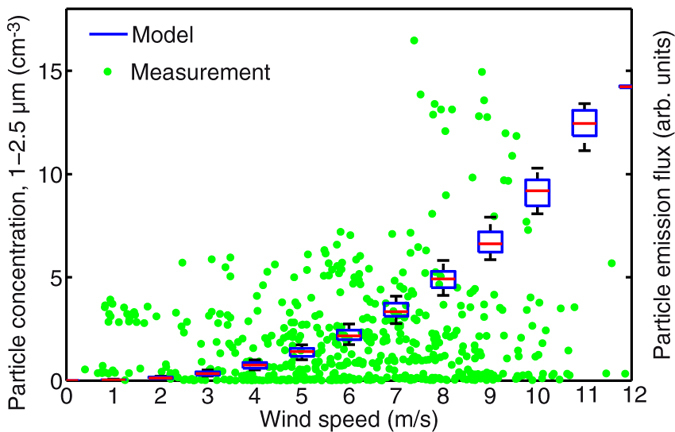
Influence of wind on particle abundance. Hourly-averaged particle concentration in the 1–2.5 μm size range. Data are compared with the empirical wind cap model of Gong *et al*.^31^ applied to the real wind measurements. The boxplots depict the magnitude of the fluctuations induced by the consideration of the Jaegle temperature correction^8^ (See text for details).

Figure 2 displays the concentration of particles in the 1–2.5 μm size range. This size corresponds to the most important mode of the sea-spray aerosols mass size distribution^28^,^29^, and has the largest direct contribution to the radiative transfer^4^. The abundance of aerosols is positively correlated with wind speed, illustrating the influence of the latter on white-cap formation and nebulization via the bursting, at the ocean surface, of bubbles originating from wave breaking^4^,^6^,^8^,^30^,^31^,^32^,^33^,^34^,^35^. Temperature also modulates the emission of particles from the ocean surface, by affecting the viscosity of water^8^. The models describing these processes primarily rely on laboratory experiments and/or large-scale ocean monitoring^4^,^6^,^8^,^18^. We propose a complementary characterization of aerosol concentration and size distribution, in the form of a continuous series of local measurements of aerosols close to the water surface in a wide variety of environmental conditions.

As shown in Fig. 2, the particle concentration remains small below a windspeed of 3 m/s, and rises beyond. This limit is consistent with the typical threshold for generating white caps^36^,^37^. We fitted our experirmental data with the model of Gong *et al*.^31^, that estimates the flux of particles as a function of the wind at 10 m altitude *u*_10_:


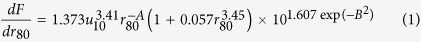


where *r*_80_ is the radius at a Relative Humidity (RH) of 80% (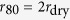), 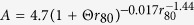, 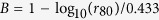, and 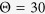. We then applied the temperature correction factor proposed by Jaegle^8^, which yields the flux:


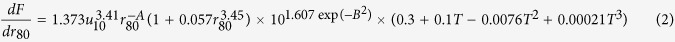


where *T* is the sea-surface temperature in °C. The dispersion of the data in the wind-temperature plane results in the spread of the model points in Fig. 2. The fit shows the consistency of this model and its temperature correction with our data.

The significant correlations between aerosols in the 1–10 μm range and local environmental parameters like wind speed and temperature (See Table S3) further suggest that these particles are mainly produced locally, as discussed above^31^.

### Organic particles

Organic particles were individually identified by the single-particle fluorescence spectrometer (GAP-SPFS, See Supplementary Information) among aerosol particles larger than 1 μm, based on their fluorescence spectrum. While the sensitivity range of the GAP-SPFS restricts the results to the larger particles, we point out that the contribution of the smaller particle sizes to the total aerosol mass is minimal, so that our results can be expected to be representative of the aerosol mass concentration.

**Figure 3:**
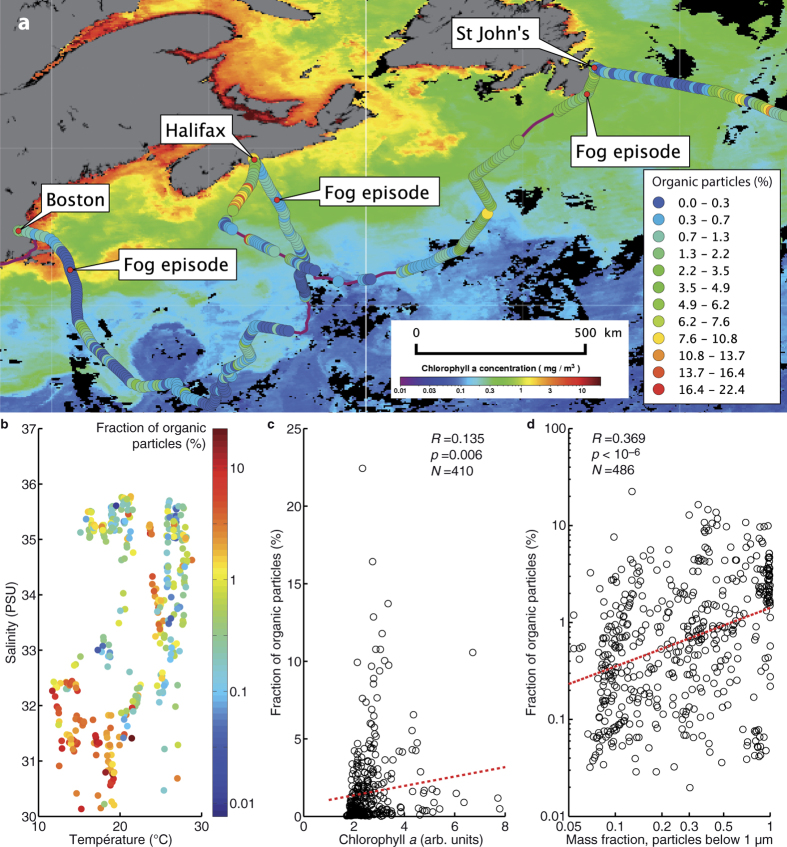
Organic aerosols. (**a**) Fluorescent particles as a fraction of the total of the single-particle counts by the fluorescence spectrometer compared to satellite-measured Chlorophyll *a* (background map, data from AQUA MODIS^26^), between Boston and St John’s. Map created with QGIS 2.12^27^. (**b**–**d**) Influence of (**b)** surface water temperature and salinity, (**c)** Chl *a*, and (**d)** the mass fraction of particles below 1 μm, on the relative abundance of fluorescent particles. Data represent hourly averages for each parameter during our expedition. *R, p*, and *N* denote Pearson’s correlation coefficient, *p* value and number of samples used for calculating the correlation, respectively. The dotted lines in (**c)** and (**d)** display the linear fits yielding the correlation coefficients displayed on the graphs.

This identification relies on the fact that UV-excited fluorescence originates almost exclusively from organic compounds, mainly from aromatic rings^38^. Although the total concentration of fluorescent aerosols locally peaks at 20’000 particles m^−3^, the averaged value over the entire expedition is 355 m^−3^ and the corresponding median is 122 m^−3^. The concentration of fluorescent particles therefore displays much larger relative variations than the total aerosol concentration. Furthermore, their spatio-temporal distributions are different. The percentage of fluorescent aerosols is negatively correlated with water temperature (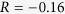, 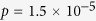) and salinity (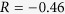, 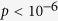) (Fig. 3b and Table S3). Although these correlations are relatively small, the associated *p* values, much smaller than 0.01, denote extremely high statistical significance. In contrast, the total aerosol concentration displays the opposite dependence (Figure S3): Warm, salty waters such as those of the Gulf Stream are associated with a higher aerosol concentration as well as a lower organic load as compared to the cold(er) water masses outside of the Gulf Stream. This decoupling is comparable to previously reported observations suggesting different origins and behaviors for organic and non-organic aerosols^17^,^18^. The influence of factors like the concentration of organic matter in the seawater, or phytoplankton blooms is likely to contribute to this decoupling.

In particular, different water masses, characterized by their temperature and salinity, exhibit very different forms of biological activity. Cold sub-polar water is usually associated with greater nutrient concentration relative to the Gulf Stream^39^, favoring the development of bacteria as well as phytoplankton^40^. Accordingly, the relative abundance of fluorescing (organic-bearing) particles is also positively correlated with the surface concentration of Chlorophyll *a* (Fig. 3a,c, Table S3). Although small (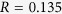) this correlation is well significant statistically (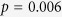).

Finally, the relative abundance of organic-bearing aerosols is highly correlated (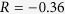, 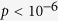) with the mass fraction of particles of 1 μm diameter and less, as measured by the optical aerosol sizer (Fig. 3d). This may be understood by considering the different emission dynamics of different particle types^17^. Organic-bearing particles are mainly film drops. In contrast, larger particles are mostly composed of sea salt emitted both as film and jet drops^6^.

**Figure 4:**
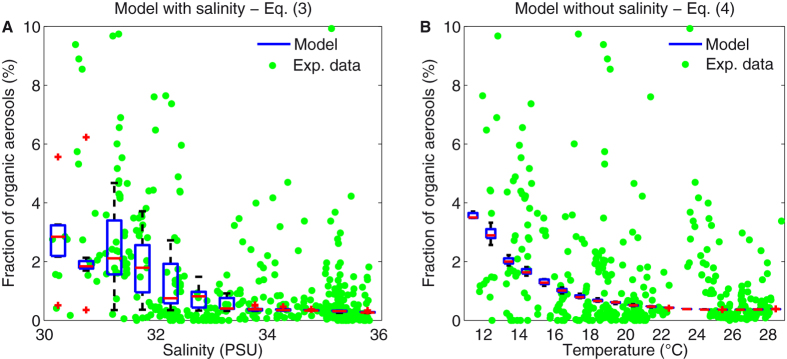
Empirical models for the fraction of organic aerosols. (**A**) Model including salinity (Equation (3)). (**B**) Model restricted to satellite-accessible parameters (Equation (4)).

Based on the time-series recorded during the expedition, we sought an empirical model capable of describing the fraction of fluorescing (organic) particles as a function of the physico-chemical parameters. We performed an analysis of variance (ANOVA), considering sea-surface temperature, salinity, Chl *a* concentration, wind, and relative humidity. As displayed in Fig. 4, up to 35% of the variance of our data can be explained by the following two-parameter model:


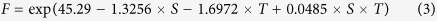


where *F* is the fraction of organic-bearing particles among the particles larger than 1 μm, in %, *S* the salinity in PSU, and *T* the sea-surface temperature in °C. Remarkably, already 25% of the variance is explained by the terms where salinity *S* is present. Conversely, considering the Chl *a* concentration, wind speed, and relative humidity did not improve the model.

Salinity is hardly accessible by remote sensing^41^, and only two satellites offer this measurement, namely the Aquarius/SAC-D^42^ and SMOS^43^. Therefore, we also sought an alternative empirical model relying only on information that is more easily accessible by remote sensing. The best model can explain up to 17% of the data variance and reads:


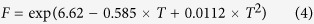


Although these empirical models are derived for summertime in the north-Atlantic ocean only, they suggest that water mass characteristics are relevant in predicting the organic load of the aerosols. In contrast, the local Chl *a* concentration appears less relevant in this regard. Such observations are in line with the reduced correlation between the dynamics of micro-organisms (e.g., plankton blooms) and the organic contents of aerosols^18^, that may be attributed to the delay between the blooms and the organic enrichment of aerosols^18^.

The high contribution of salinity to Equation (3) may seem surprising, as it is not expected to influence the biological activity, nor the nebulization mechanism. We may speculate that salinity plays here the role of a tracer of the water masses^44^. Its domination over sea-surface temperature in that regard could be related to the larger short-term variability of temperature, especially due to the interactions with the atmosphere. Furthermore, as the latent heat of vaporization of water (2265 kJ/kg) is larger than the specific heat of water (4.18 kJ/kg/K), less energy is needed to significantly affect the water temperature than to influence the salinity. Finally, salt is known to assist the degradation of organics during particle aging^45^,^46^. It can therefore be expected that high salinity reduces the organic concentration, hence the associated particle fluorescence.

For this reason, our empirical models display more dynamics, and explain a larger fraction of the variability, than previously proposed ones that were mainly based on Chl *a* concentration^21^,^22^,^47^,^48^,^49^. This may also explain the controversy about the validity of using Chl *a* as a proxy for organic sea spray aerosols as proposed by Quinn *et al*.^17^ in spite of contradictory results^4^,^17^. Further modelling would require a better consideration of particle transport, although as discussed above we expect that the local contribution is high in our data.

Our data therefore provide relationships between environmental parameters (especially temperature, salinity and wind speed) that can be easily monitored, *in-situ* or remotely via satellites, and the aerosol abundance as well as their organic content. The use of synoptic proxies for the estimation of large scale distribution and abundances of organic aerosols will help to evaluate their impact on the Earth’s radiative balance, especially their indirect effect through nucleation activity. In particular the parameterization of the organic aerosol fraction allows differentiating the effects of various particle types (that are defined by their composition) on the radiative balance, including their impact on nucleation.

### Local dynamics

Nevertheless, the variability of the fraction of organics-bearing particles displayed in Fig. 4 is quite large, due to the effect of the local variability in environmental conditions, which add to the global trends. Such events include (i) the crossing of the cold water mass of the Labrador Current, south of Halifax (See Fig. 3a), where the fraction of organic-bearing aerosols can represent up to 20% of the total particles, (ii) three fog episodes (Figs 1a, 3a, and Table S1) where the total particle number concentration significantly rises without any discernible impact on the organic particle concentration, and (iii) the intersection of three eddies by the solar-powered ship (See Table S1 and Figure S6). In particular, a cold-core eddy was characterized by significantly (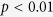) lower temperature and salinity, and higher dissolved oxygen and Chl *a* concentration. Simultaneously, the concentration of particles increased in the 3–4 μm range and decreased for sub-μm particles. Therefore, the overall relationships between environmental parameters with both inorganic and organic aerosols significantly deviate from outside the eddy.

### Organic (fluorescent) aerosol particle speciation

**Figure 5:**
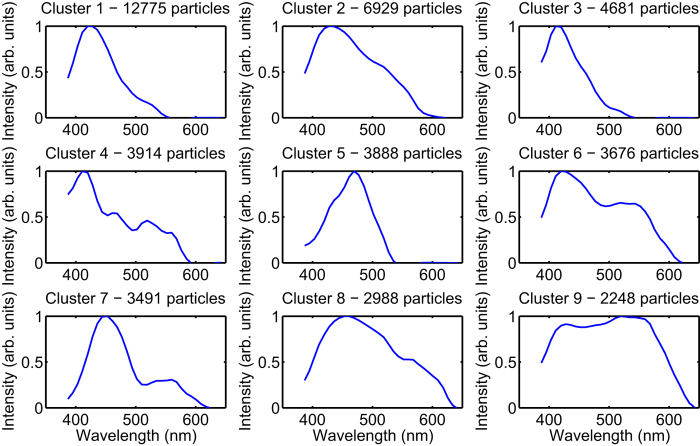
Organic particle speciation. Fluorescence spectra of the main self-identified particle clusters, ordered by decreasing abundances.

To gain a qualitative insight into the organic particles, we grouped the individual particles into clusters of similar fluorescence spectra as measured by the GAP-SPFS. Figure 5 displays the spectra of the nine most abundant clusters, which individually account for at least 7.5% of the fluorescent particles collected at sea. Together, these clusters account for 85% of the fluorescent particles. It should be noted that spectra could only be measured for particle sizes in the coarse mode, in the size range from 1–30 μm, which allows detection of microorganisms and water sprays in this size range, but not organics from the particles in the Aitken or accumulation modes.

The nine identified families of spectra are well distinguishable, and were observed consistently throughout the entire campaign. A direct assessment of the composition of each aerosol particle based on these characteristic spectra is often impossible, since each aerosol particle is constituted of a complex mixture of fluorescent components^20^. A dominant feature for the majority of the analysed particles (in particular clusters 1 and 2) is a broad blue emission peaking around 420 nm, while excited at 337 nm. This emission is often related to the fluorescence of coenzymes like hydrogenated Nicotinamide adenine dinucleotide (phosphate) (NAD(P)H) and pyridoxamine, as well as cellulose, chitin and pteridine compounds^38^. These fluorophores dominate the reference spectra of the organisms measured by the GAP-SPFS (Figure S5). Besides the vitamin B6-related pyridoxamine, vitamin B2-related compounds like riboflavin, flavin mononucleotide (FMN) and flavin adenine dinucleotide (FAD) are often responsible for broad and weaker features around 550 nm, as observed in clusters 2, 4, 6 and 7. The fluorescence of aromatic amino acids (tryptophan, tyrosine, phenyl-alanine) is not accessible here, since they do not significantly absorb at 337 nm.

The broad fluorescence spectra, characteristic of clusters 6 and 9 are reminiscent of spectra from humic-like substances (HULIS, See also Figure S5), which comprise more than 100 chemical species^50^. They constitute an essential component of natural organic matter, both in soil and in water. These biogenically and terregeneous-derived, heterogeneous organic substances constitute less than 3% in the open ocean, whereas they represent 50–80% of the total dissolved organic matter in freshwaters^51^. In accordance with this, few HULIS were observed, and indeed these represent just 0.2% of the fluorescent aerosols. Most of the HULIS particles were detected close to the coast, which is consistent with the terrestrial origin of these aerosols.

The temporal series of all main clusters except HULIS are highly correlated (Figure S4), showing that the corresponding fluorescing (organic-bearing) species “cocktails” behave similarly in time, space, and for various atmospheric and oceanic conditions. Such observation is consistent with the fact that organic carbon displays similar properties in aerosols at various locations^17^. Despite the relatively constant concentration of total dissolved organic carbon in the North Atlantic (50–80 μM^52^), recent analyses highlighted the fact that several thousand different organic compounds are simultaneously present in surface waters^53^, some of which can be produced and transformed as a result of biological activity^50^. As for the aerosols, organic compounds of different molecular sizes are present in solution^52^, ranging from low to high molecular weight and colloidal material, contributing to the size continuum between dissolved and particulate organic matter^54^. Our results confirm that, at least in the North-Atlantic and in Summer, a homogeneous “cocktail” containing the entire spectrum of dissolved organic compounds, including micro-organisms, is emitted simultaneously and constitutes about 2–5% of the total aerosol mass^4^.

In order to better assess the composition of self-referenced fluorescence clusters, we are currently developing a new dedicated instrument, which will selectively sort particles exhibiting the same spectrum^55^. This selective sampling will allow further laboratory chemical analyses in the laboratory and enable a look-up table for the future campaigns to be created.

## Conclusion

In summary, this study enabled to characterize the contrasted spatial and temporal dynamics of total and organic marine aerosols, respectively. We propose an empirical parameterization of the fraction of organic aerosols, based exclusively on the salinity and temperature of the water masses encountered during the measurement campaign. Such relationships may provide a deeper insight into the mechansisms of particle formation. It also offers a unique opportunity to estimate the total and organic aerosol load over the ocean from easily-measured ocean and atmosphere physico-chemical parameters. The distinction between particle types with different direct and indirect impacts on albedo could help refinig estimations of the influence of aerosols on the Earth’s radiative balance, in particular their ability to promote nucleation.

The analysis of the fuorescent spectrum of individual particles revealed that a very rich mixture of organic aerosols appear to be emitted simultaneously. A detailed characterization would therefore require the chemical analysis of individually-sampled particles. It would provide an even better characterization of the organic aerosols, e.g., by allowing the measurement of the particle enrichment in organics during their evolution^4^. Similarly, taking into account the sea surface microlayer^56^ and the role of its surfactants on waves and aerosol formation^24^,^57^ would allow a more precise analysis of the link between the seawater composition and the aerosol production^58^,^59^,^60^,^61^,^62^,^63^,^64^,^65^,^66^.

## Additional Information

**How to cite this article:** Kasparian, J. *et al*. Assessing the Dynamics of Organic Aerosols over the North Atlantic Ocean. *Sci. Rep.* **7**, 45476; doi: 10.1038/srep45476 (2017).

**Publisher's note:** Springer Nature remains neutral with regard to jurisdictional claims in published maps and institutional affiliations.

## Supplementary Material

Supplementary Information
