# Supplementary material for: Assessing the Dynamics of Organic Aerosols over the North Atlantic Ocean
Source: Sci Rep. 2017 Mar 31;7:45476. doi: 10.1038/srep45476 (PMC5374472; doi:10.1038/srep45476)
Supplement: Supplementary Information [file srep45476-s1.pdf]

Assessing the Dynamics of Organic Aerosols over the North Atlantic Ocean Supplementary Information

Jérôme Kasparian[1,*], Christel Hassler[2,3], Bas Ibelings[2,3], Nicolas Berti[1], Sébastien Bigorre[4], Violeta Djambazova[2], Elena Gascon-Diez[2], Grégory Giuliani[2], Raphaël Houlmann[1], Denis Kiselev[1], Pierric de Laborie[2], Anh-Dao Le[2,3], Thibaud Magouroux[1], Tristan Neri[2], Daniel Palomino[2,3], Stéfanie Pfändler[2], Nicolas Ray[2], Gustavo Sousa[1], Davide Staedler[1,5], Federico Tettamanti[5], Jean-Pierre Wolf[1], Martin Beniston[1,2, 3]

1. Université de Genève, Group of Applied Physics, Chemin de Pinchat 22, CH1211 Geneva 4, Switzerland
2. Université de Genève, Institute for Environmental Sciences, 66 Boulevard Carl Vogt, CH 1211 Geneva 4
3 Université de Genève, Department F.-A. Forel for Environmental and Aquatic Sciences, 66 Boulevard Carl Vogt, CH 1211 Geneva 4,
Switzerland
4. Woods Hole Oceanic Institute, 86 Water St, Woods Hole, MA 02543, USA
5. TIBIO SagL, via alla Valle I I, 6949 Comano, Switzerland

* Corresponding author. E-mail: jerome.kasparian@unige.ch

Supplementary Materials and methods

Table S1. Expedition route and main events.

Milestone	Date	Distance travelled (km)	Latitude (°)	Longitude (°)
Boston stopover	04/07/2013	0	42.355	-71.044
Fog episode 1	05/07/2013	152	41.626	-69.61
	05/07/2013	291	40.422	-69.19
Cold-core eddy	08/07/2013	942	36.777	-64.914
	09/07/2013	1067	37.466	-64.544
Fog episode 2	15/07/2013	1955	43.207	-62.734
	15/07/2013	1994	43.519	-62.931
Halifax stopover	15-21/07/2013	2129	44.646	-63.569
Warm-core eddy 1	25/07/2013	2632	41.446	-62.39
	26/07/2013	2800	40.736	-60.857
Warm-core eddy2	27/07/2013	2902	40.85	-59.726
	27/07/2013	3055	41.279	-58.029
Fog episode 3	31/07/2013	3842	46.614	-53.066
	01/08/2013	3873	46.852	-52.87
St John's stopover	01/08/2013	3959	47.563	-52.707

Table S2: Instruments and Data available during the PlanetSolar Deepwater campaign

Device	Measured parameters	Specifications	Remarks
Optical Aerosol sizer	Aerosol concentration and size distribution	31 size classes.<br>250 nm — 32 µm<br>+ nanoparticle counter (25-300 nm)	6 s resolution, hourly averages
Single-particle fluorescence spectrometer	Aerosol concentration and composition	UV fluorescence spectra of individual particles.	Continuous measurement, hourly average
CTD profiles	Vertical profiles of water physical and chemical parameters	Pressure, Temperature, conductivity, salinity, dissolved oxygen, phycoerythrin, chlorophyll *a*	49 vertical profiles, 0 – 200 m<br>1s resolution
Ferrybox	Surface water physical and chemical parameters	Temperature, conductivity, salinity, dissolved oxygen, phycoerythrin, chlorophyll *a*	Continuous measurement, 1 s resolution, hourly average
Weather station	Weather parameters		Continuous measurement, hourly average
Ship data recorder (Datalogger)	Weather and ocean climatic parameters and routing information	GPS position, speed over water, wind, see and air temperature…	30 s resolution, hourly average
AQUA MODIS satellite (5,6,7)	Remote sensing of sea-surface parameters	Sea-surface temperature (SST), Chlorophyll *a* (Chl *a*)	Use of the 8-day composite data

Table S1 summarizes the expedition route and specific events. Along this trajectory, dynamic, chemical, and biological characteristics of the ocean and the overlying atmosphere were continuously monitored using several instruments, as summarized in Table S2. Aerosols have been characterized by two complementary instruments operating in parallel. The two instruments are designed to sample air on the front side of the deck of the ship with a common access, shielded from wind by a cap.

Single particle fluorescence spectrometer

A single-particle fluorescence spectrometer (FPSF) was developed specifically for the present campaign in order to identify in real-time the aerosol particles present in

the marine atmosphere. As sketched in Figure S1, the identification was achieved by measuring the scattering properties and the fluorescence spectrum of individual aerosol particles embedded in the atmospheric flow. In order to achieve this, the air was sampled at 60 L/min 5 m above sea level, concentrated by a 10x aerodynamic lens, and then focused by an optimally-designed sheath nozzle in a narrow stream of 500 µm diameter (1). This stream crosses infrared lasers for the scattering measurements, as well as a UV laser for exciting the fluorescence (2,3) that is detected by a multi-angle laser scattering module (4). This procedure also allows measuring simultaneously the optical size of each particle. Active “on the fly” modulation of the laser intensity is also applied so that saturation is prevented and particles whose sizes range from less than 1 µm to 60 µm can be analyzed. Scattering signals are then further used to trigger the pulsed UV laser (337 nm), which excites the fluorescence of the same individual particle. The fluorescence is retrieved by a dedicated reflective lens and analyzed with a 32-channel spectrometer from 360 nm to 650 nm. This spectrometer is based on a multi-anode photomultiplier (Hamamatsu H7260-03) and specifically-designed acquisition electronics.

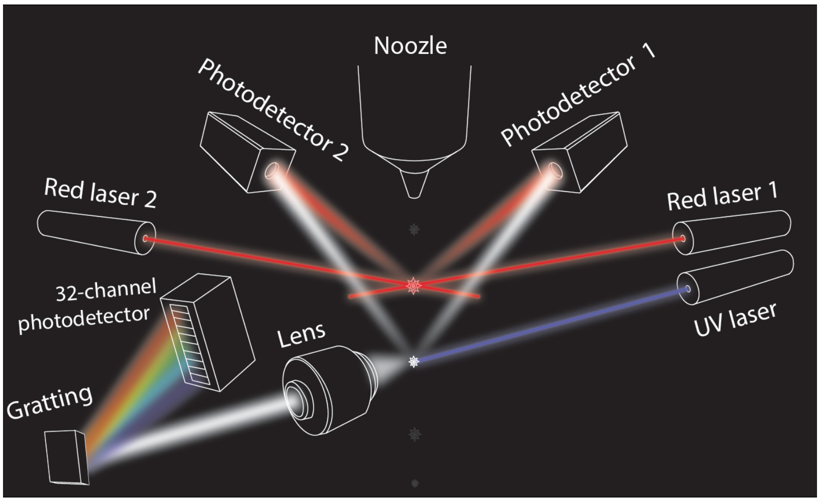

Figure S1. Principle of the single-particle aerosol spectrometer. The instrument allows measuring simultaneously multi-angle elastic scattering and the UV-Vis fluorescence spectra of individual aerosols

The particles were sorted into clusters displaying similar fluorescence spectra, in a self-referenced procedure. A particle with a spectrum matching a previously-identified species is considered as a positive identification of the particle. Conversely, a particle matching no previously-identified cluster spectrum is considered as the prototype of a new cluster. The procedure was repeated after averaging the spectrum of each cluster over all its particles, in order to avoid overweighting the spectrum of the first particle within each cluster. The population of each cluster was then accumulated over hourly intervals.

This self-referencing method avoids bias from *a-priori* selected reference spectra, to which experimental spectra are compared. This method was successfully applied

with a similar aerosol fluorescence spectrometer developed at Yale (3), in the context of other environments, like urban pollution.

In parallel, we measured in the laboratory with the same instrument the fluorescence signatures of aerosols containing chemical compounds or organisms likely to be found in the ocean during the expedition. For this purpose, we vaporized them into a spray with a size distribution as close as possible to that measured during the campaign, and measured their fluorescence spectra in the GAP-SPFS operating in its standard mode. These spectra were averaged over several tens to several hundreds of individual particles per species. The inter-particle variability for each species was then used as a reference for the variability allowed in the cluster identification procedure.

Optical aerosol sizer

An optical aerosol sizer (Grimm 1.109) continuously sampled the air 5 m above sea level and measured the aerosol size distribution by optical scattering. It sorted the particles in 31 classes ranging from 250 nm to 32 µm. Note that this on-line measurement concerns the real aerosol size, as particles are not dried prior to, nor during, the measurement. The instrument was equipped with a nanoparticle sensor (Grimm Nanocheck 1.365) measuring the number concentration of particles in the range 25 – 300 nm. This instrument samples 1.5 L/min through a tube that is 4 mm in diameter and ~1 m long. Measurement were recorded every 6 s, with a subsequent hourly averaging.

Conductivity, Temperature, Depth (CTD) vertical profiles

49 CTD profiles using an Idronaut Ocean Seven 316 Plus CTD, equipped with sensors for pressure, temperature, conductivity and oxygen, a Trilux sensor, which measures Chl-*a,* phycocyanine and phycoerythrin fluorescence and functions as a nepheolometer, and a Licor LI-193SA quantum sensor. This CTD was lost at sea (July 14th) and replaced by a Minos X, equiped with conductivity, temperature and pressure sensors (C•Xchange, T•Xchange, P•Xchange, respectively) for the remainder of the cruise.

Ferrybox

A Ferrybox continuously monitored the surface water by pumping water from approximately 1 m below sea surface at the rear of the ship. The water was analyzed in an Idronaut Ocean Seven 316 Plus, equipped with the same sensors as the CTD described above. The Chlorophyll *a* measurement was tested for consistency against satellite data (See Data validation below)

Datalogger

The ship was equipped with a driving assistance system and various sensors that were continuously logged. In addition to the technical parameters specific to the ship’s navigation and performance (GPS location, heading, speed over ground and water, propulsion power), the recorded parameters included solar irradiance, air

temperature, pressure, and wind speed and direction as well as sea temperature measured 1 m below the surface.

Weather station

In addition to its own independent measurements of atmospheric parameters, the ship was also equipped with a weather station (Batos, MeteoFrance), which continuously monitored wind speed, atmospheric pressure, air temperature and relative humidity, as well as the sea surface temperature. It also independently recorded the ship's trajectory, heading and speed.

Satellite data

We used the AQUA MODIS satellite 8-day composite Sea-Surface Temperature (SST) and chlorophyll *a* concentration data (5,6,7,8) to both request specific positioning of the ship during the expedition and also to cross-check the locally measured environmental parameters.

Data validation

All instruments were synchronized with a master clock for the entire duration of the campaign. The location of each measurement was determined according to this master clock, associated with the ship's trajectory data. After the validation and quality control specific for each instrument, data were averaged over hourly time intervals.

In a second step, we checked the consistency between the instruments whenever possible. This concerns in particular the atmospheric conditions (temperature, relative humidity, wind) from the ship data-logger and the Batos weather station; total aerosol concentrations from the GAP-SPFS and Grimm aerosol detectors, over the 1 µm – 32 µm size range where both are sensitive (e.g., $R = 0.69$, $p < 10^{-6}$ for the 1-2.5 µm size range); and marine parameters (sea surface temperature, Chlorophyll *a*) between the Ferrybox and AQUA-MODIS data ($R = 0.97$, $p < 10^{-6}$ and $R = 0.45$, $p < 10^{-6}$, respectively, see also Figure S2). However, as the measurement conditions are not identical, we did not use the data of Figure S2 as a calibration of the Ferrybox measurement of Chl *a*, that are provided as arbitrary units.

The consistency with the Ferrybox with the first layers of the CTD profiles was also checked for SST ($R = 0.999$, $p < 10^{-6}$), and salinity ($R = 0.73$, $p < 10^{-6}$).

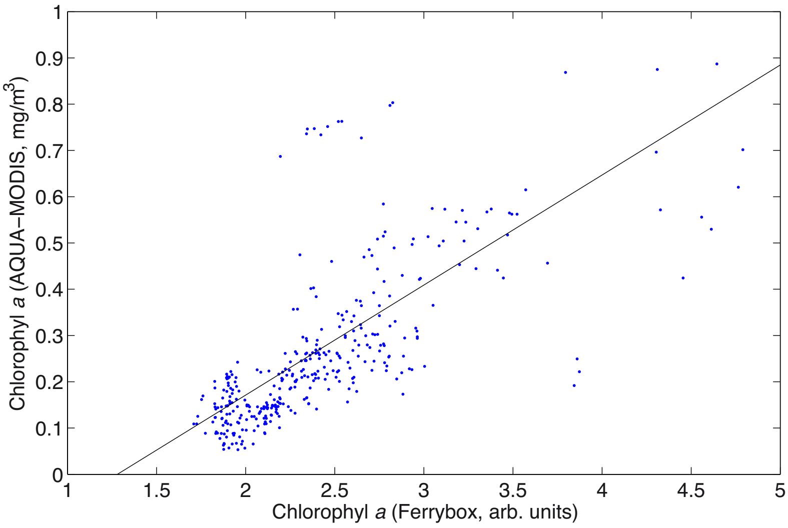

Figure S2. Comparison of Chlorophyll *a* measured by the on-board Ferrybox with the AQUA-MODIS Chl *a* satellite data during the expedition.

Supplementary Figures and Tables

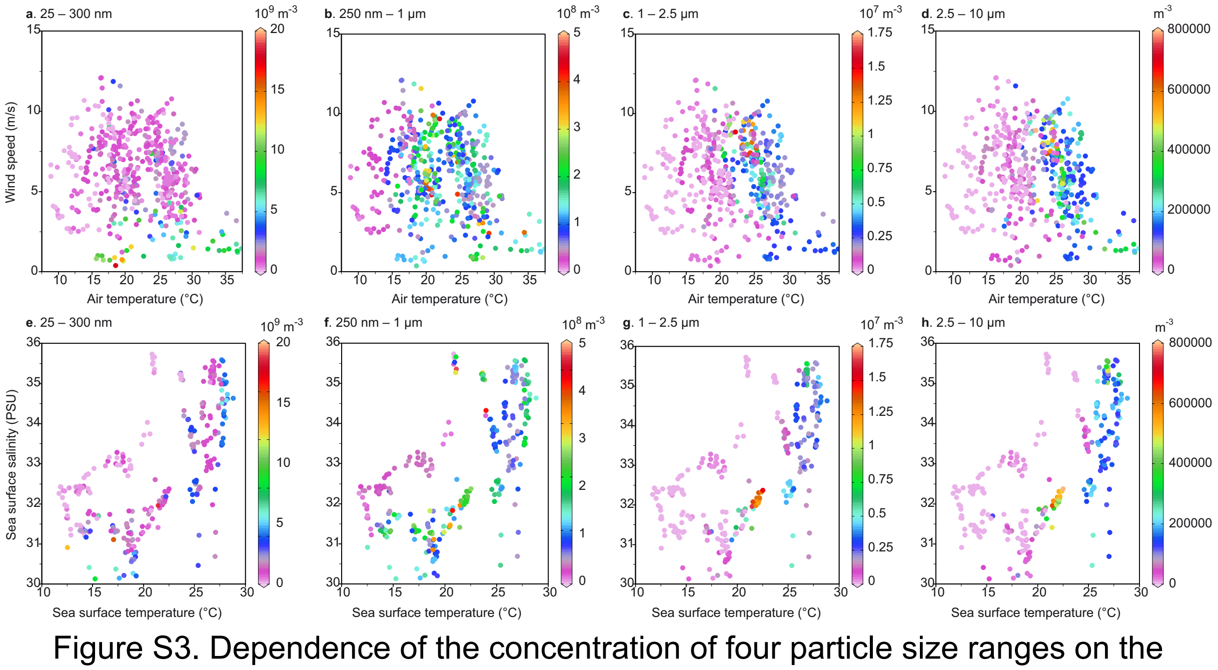

Figure S3. Dependence of the concentration of four particle size ranges on the atmospheric conditions (air temperature and wind speed, **a** – **d**) and oceanic conditions (salinity and sea surface temperature, **e** – **h**). Data represent hourly averages for each parameter during the expedition.

Table S3: Statistical comparison between sea surface properties and particle abundance and size. Pearson's correlation coefficient, *p* value and number of samples used for calculating the correlation are provided by hourly averages of the parameters collected by the Ferrybox (temperature, conductivity, and Chl *a*) and aerosols in the 1 – 10 µm size range as measured by the optical aerosol sizer. Statistically significant positive correlations are highlighted in red, negative ones in blue.

	**1 — 10 µm**	**Fraction of organic particles**
**Water temperature (◦C)**	**0,36**<br>$< 10^{-6}$<br>*441*	**-0.16**<br>1.5×10^{-5}<br>*741*
**Salinity (PSU)**	0.08<br>0.17<br>*283*	**-0.46**<br>$< 10^{-6}$<br>*410*
**Chl *a* (FSU)**	-0.06<br>0.30<br>*284*	**0.13**<br>7.7×10^{-3}<br>*411*
**Atmospheric pressure (bar)**	**-0.49**<br>$< 10^{-6}$<br>*441*	**-0.18**<br>$< 10^{-6}$<br>*741*
**Air temperature (°C)**	**0.48**<br>$< 10^{-6}$<br>*441*	**-0.15**<br>2.5×10^{-5}<br>*741*
**Relative humidity (%)**	**0.18**<br>9.8×10^{-5}<br>*441*	**0.13**<br>5.6×10^{-4}<br>*741*
**T/S Ferrybox**	**0.44**<br>$< 10^{-6}$<br>*283*	**-0.21**<br>1.8×10^{-5}<br>*410*

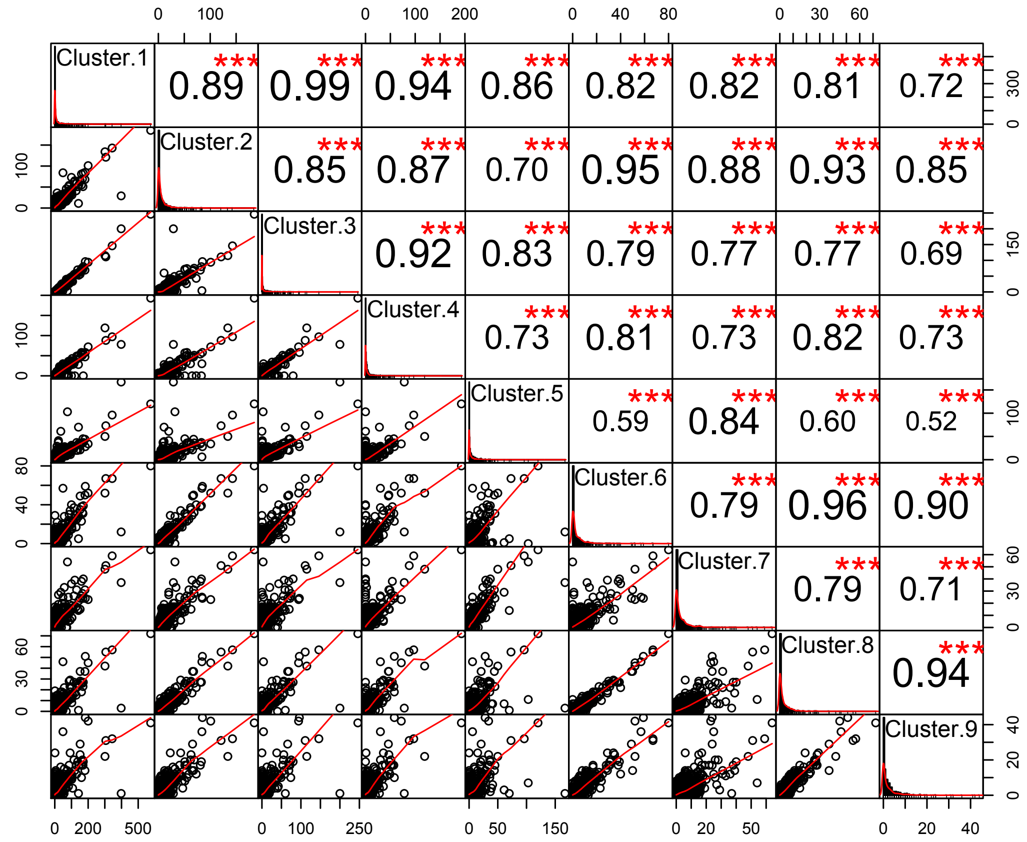

Figure S4. Temporal correlations between the 9 main self-identified clusters of organic particles.

In order to verify the discrimination capability of the GAP-SPFS for some biological samples that may be present in the ocean, we performed laboratory tests using an atomizer and reference organisms. Figure S5 clearly demonstrates the capability of recognizing the characteristic broad spectrum from humic acid or the yellow peaked fluorescence of vitamin B2 (Riboflavin). The cyanobacterium Synechococcus and the planktonic diatoms Chaetoceros, exhibit, among others, the characteristic fluorescence of NAD(P)H in the blue and some Flavin related components in the yellow/red. It is interesting to notice that both the species and the nutriment condition (e.g. Iron rich or Iron poor) influence the fluorescence spectra. This was also previously observed for other bacteria (2)

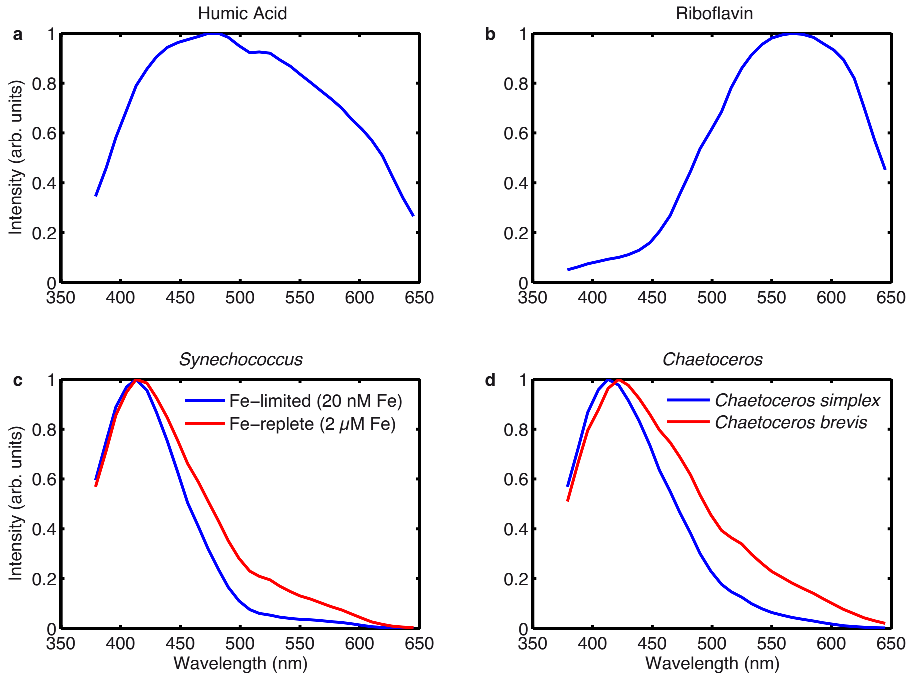

Figure S5. Reference spectra of marine species and organisms, measured in conditions representative of the campaign and with the same instrument. All organisms have been measured in saltwater.

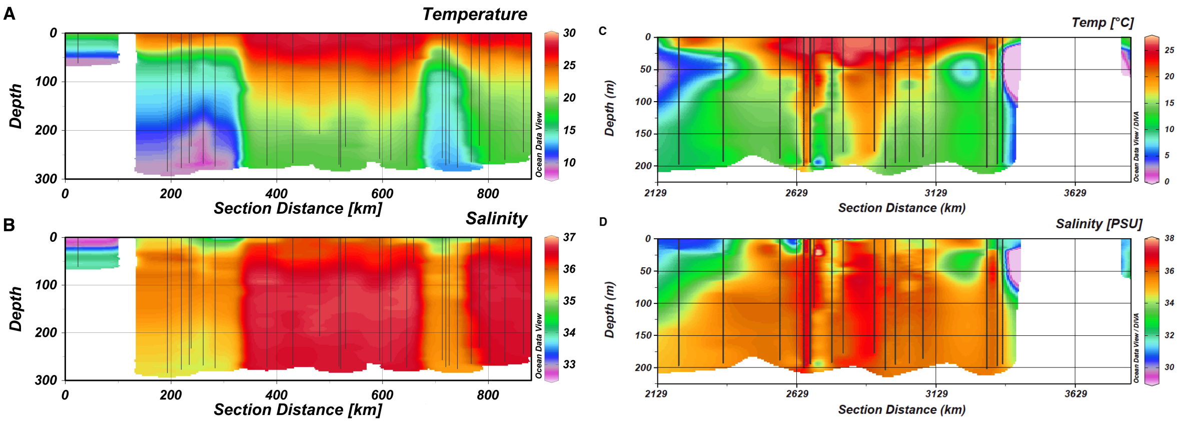

Figure S6. Variation of temperature and salinity along the cruise track from Boston to Halifax (**A**, **B**) and from Halifax to St Johns, Newfoundland (**C**, **D**). The location of the cold-core and the warm-core eddies are indicated. Data is plotted using Ocean Data View 4.5 (9).

Supplementary references

1 Kiselev, D., Bonacina, L., Wolf, J.P., A flash-lamp based device for fluorescence detection and identification ofindividual pollen grains, *Rev. Sci. Instrum.* **84**:033302 (2013). DOI: 10.1063/1.4793792

2 Hill, S.C., *et al.* Real-Time Measurement of Fluorescence Spectra from Single Airborne Biological Particles, *Field analytical chemistry and technology* **3**:221–239 (1999). DOI: 10.1002/(SICI)1520-6521(1999)3:4/5<221::AID-FACT2>3.0.CO;2-7

3 Pan, Y., *et al.* High-speed, High-sensitivity Aerosol Fluorescence Spectrum Detection Using 32-Anode PMT Detector, *Rev.Sci.Inst.* **72**:1831-1836 (2001). DOI: 10.1063/1.1344179

4 Bonacina, L., Kiselev, D., Wolf, J.P. Measurement Device and Method for Detection of Airborne Particles, European Patent EP 12167800.7 and US Patent 2013/0301047A1 (2013)

5 NASA Goddard Space Flight Center Ocean Ecology Laboratory, Ocean Biology Processing Group. SeaWiFS Ocean Color Data. doi:10.5067/ORBVIEW-2/SEAWIFS_OC.2014.0 (2014)

6 NASA, Ocean Color Feature, http://oceancolor.gsfc.nasa.gov/cms/ (Date of access: 06/10/2016)

7 Moradi M. & Kabiri K. Spatio-temporal variability of SST and Chlorophyll-a from MODIS data in the Persian Gulf, *Marine pollution bulletin* **98**:14 (2015). DOI: 10.1016/j.marpolbul.2015.07.018

8 Werdell, P. J., & Bailey, S. W. An improved bio-optical data set for ocean color algorithm development and satellite data product validation. *Remote Sensing of Environment* **98**:122-140 (2005). DOI:10.1016/j.rse.2005.07.001

9 Schlitzer, R. Ocean Data View (version 4.5, 2012). Available at: http://odv.awi.de (Date of access: 06/10/2016)
